# Allergenius, an expert system for the interpretation of allergen microarray results

**DOI:** 10.1186/1939-4551-7-15

**Published:** 2014-06-25

**Authors:** Giovanni Melioli, Clive Spenser, Giorgio Reggiardo, Giovanni Passalacqua, Enrico Compalati, Anthi Rogkakou, Anna Maria Riccio, Elisabetta Di Leo, Eustachio Nettis, Giorgio Walter Canonica

**Affiliations:** 1 Dipartimento di Medicina Interna, Università di Genova, Largo Rosanna Benzi 10, 16132 Genova, Italy; 2 Logic Programming Associates, Studio 30, Royal Victoria Patriotic Building, Trinity Road, London SW18 3SX, UK; 3 Unità di Biometria, Mediservice S.r.l., Via Matteotti, 43/B - 20864 Agrate Brianza (MI), Italy; 4 U.O. Allergologia e Immunologia Clinica, Policlinico, Università di Bari, Piazza Giulio Cesare, 11 - 70124 Bari, Italy

**Keywords:** 3 TO 10 DIFFERENT ImmunoCAP ISAC, Allergen microarray, Artificial intelligence, Expert system, ISAC interpretation, Genuine component, Cross-reactive component, Computer supported diagnosis

## Abstract

**Background:**

An in vitro procedure based on a microarray containing many different allergen components has recently been introduced for use in allergy diagnosis. Recombinant and highly purified allergens belonging to different allergenic sources (inhalants, food, latex and hymenoptera) are present in the array. These components can either be genuine or cross-reactive, resistant or susceptible to heat and low pH, and innocuous or potentially dangerous. A large number of complex and heterogeneous relationships among these components has emerged, such that sometimes these interactions cannot be effectively managed by the allergist. In the 1960s, specialized languages and environments were developed to support the replacement of human experts with dedicated decision-making information systems. Currently, expert systems (ES) are advanced informatics tools that are widely used in medicine, engineering, finance and trading.

**Methods:**

We developed an ES, named Allergenius ®, to support the interpretation of allergy tests based on microarray technology (ImmunoCAP ISAC ®). The ES was implemented using Flex, a LPA Win-Prolog shell. Rules representing the knowledge base (KB) were derived from the literature and specialized databases. The input data included the patient’s ID and disease(s), the results of either a skin prick test or specific IgE assays and ISAC results. The output was a medical report.

**Results:**

The ES was first validated using artificial and real life cases and passed all in silico validations. Then, the opinions of allergists with experience in molecular diagnostics were compared with the ES reports. The Allergenius reports included all of the allergists’ opinions and considerations, as well as any additional information.

**Conclusions:**

Allergenius is a trustable ES dedicated to molecular tests for allergy. In the present version, it provides a powerful method to understand ISAC results and to obtain a comprehensive interpretation of the patient’s IgE profiling.

## Background

The availability of microarrays (MA) of recombinant or highly purified allergenic components has significantly improved the study of the IgE repertoire [1]. This is of particular relevance in poly-sensitized patients [2], in which up to 50% of the treatments can benefit from this approach [3]. Moreover, molecular analysis of the sensitizations allows for the refinement of the prescription of a specific immunotherapy [4,5] and a prediction of an outcome for the immunotherapy treatment [6]. However, the interpretation of the MA reports is sometimes a difficult task [7]. Indeed, according to their molecular characteristics, the components can be divided into inhalants or food, seasonal or perennial, genuine or cross-reactive, heat, pH and peptidase resistant or susceptible, innocuous or dangerous, causing local or systemic effects, etc. The relationships between all of these characteristics are complex [8]. For this reason, the professionals need to be trained, and a comprehensive approach in preparing and explaining the reports is required [7]. In recent years, two generations of allergen MA (the first with 103 and the second with 112 components) were developed (ImmunoCAP ISAC®, Thermo-Fisher, Milano, Italy). These tools have been extensively validated in different clinical settings [9-11]. The recent 112 component version may include a specialized report, generated by the X-Plain ® software, that explains the nature of the positive components. However, although this is sufficient for the management of a majority of the cases, it is not completely suitable for complex patients.

The information needed to interpret an ISAC report is available in specialized literature, and the knowledge in this field is rapidly progressing. Computer-supported diagnosis (CSD) tools, based on expert systems (ESs), were developed in the 1960s and have been used in the past to support clinical routines [12-16]. At present, ESs are deeply integrated in many informatics tools, including medicine, finance, trading, engineering, social network analysis and web research engines. By definition, ESs are a part of the artificial intelligence (AI) world; however, because they belong to the knowledge-based systems, ESs are not considered auto-learning tools. They are intended to act as human experts consulted to obtain advice, suggestions and recommendations on problems that fall within the experts’ knowledge [17]. Thus, during the development of an ES, the external consultants indicate both the objects that are involved and the rules that govern these objects [18]. Certainly, the continuous update of knowledge, based on the experts’ experience and on scientific improvements, allows for a real-time maintenance of the objects and rules.

Practically, ESs are composed of three independent structures: the experts’ knowledge base (KB), the inferential engine (IE) that manages the set of rules, and the interface with the outside world (I/O). The IE works even if the KB is significantly modified, allowing the KB to be largely independent from the IE. For all of these reasons, MA interpretation seems to be a natural challenge that can benefit from an artificial intelligence approach. To design and implement such an ES, we first analyzed how molecular allergy experts extract the relevant data from an ISAC report. Additionally, we identified what additional information that is not immediately available in the report can be obtained from a comprehensive interpretation of the ISAC results. In this report, we describe the structure and validation procedures of an ES developed for the interpretation of ISAC results.

## Methods

### Collection of information

Definitions were used according to the literature [7,8]. The KB and the rules were derived from published data [19], the authors’ experience [4,10,11] and the experts’ opinions. Specific information on the components was obtained from the Allergome [20], Allergen online [21], RCSB [22], Uniprot [23] and the WHO/IUIS Allergen Nomenclature websites [24,25]. The cross-reactive components were ordered into families [26]. The allergen- or component-associated clinical pictures, such as the pollen-food syndromes were also considered [19,27,28]. Finally, the rules for the classification of the phenotypes [29] and strategies for the prescription of allergen specific immunotherapies [3-6,30-32] were also implemented.

### Construction of the ES structure

The ES code was written using Flex, an advanced Knowledge Specification Language (LPA, London UK). This ES environment was chosen because it is suited to symbolic problems, rather than numerical ones. Flex is written in plain English, which allows non-professional programmers to update the code. Functionally, the ES is based on frames (representing the KB), rules that drive the system itself and I/O structures.

### Knowledge bases

The frames containing facts were based on a classic hierarchical structure. They contained the 112 ISAC components (Phl p1, Der p 2, etc.) and their attributes, such as class (inhalant, food, venom, latex), group (mites, trees, etc.), origin (Betula v., Olea e., etc.), nature (genuine, cross-reactive), family (PR-10, nsLTP, profilin), sub-family (prolamin, etc.) and molecule (alpha-amylase, gliadin-omega-5, etc.). Additionally, the component frequency, median value, maximum value and 97.5 percentile (in ISU), resistance to heat, acid pH and peptidase and comment(s) including specific references were also added. This frame structure is extremely efficient because it can be easily modified to update or change the information without affecting the governing rules.

### Input/output structures

This interface allows for the addition of the patient's personal and clinical data, including the results of the ISAC and other assays, such as SPT and sIgE, to the ES. The language (English or Italian) and the level of detail in the report (basic or advanced) can also be defined by the user.

### Rules

More than 700 different rules were implemented to mimic the experts’ opinions on a complex ISAC result. The rules were classified as top, intermediate and low level rules, on the basis of the impact that each rule has on the decision tree. An example of these rules is presented in Table 1.

**Table 1:** Rules implemented in the expert system Allergenius

Top level rules (examples)	
	1. IgE specific for a given allergen or component are signs of sensitization. Specific IgE and relevant clinical signs are indicative of an allergy.
	2. Inhalant allergens are either genuine or cross-reactive.
	3. Microarray allergens are classified as inhalants, food, contacts or venoms.
	4. Allergens are classified as innocuous or potentially dangerous [8].
	5. Components are classified as recombinant molecules (without glycidic chains, such as rPhl p 1) or highly purified extractive molecules (containing glycidic chains, such as nCyn d 1).
	6. Relationships between microarray components and allergens:
	a) A positive component is generally associated with the positivity of the relevant allergen (e.g., der p 1 and D1 are positive).
	b) Negative components are generally associated with the negativity of the relevant allergen (e.g., Der p 1, Der p 2, Der p 10, Der f 1 and Der f 2 are negative, as well as D1).
	c) Negative components are sometimes associated with a positive allergen (e.g., Der p 1, Der p 2, Der p 10, Der f 1 and Der f 2 are negative and D1 is positive). However, the frequency of these cases is known [10], and the allergen score is generally very low.
	d) If one or more components are positive but the relevant extractive allergen is negative, the rare, and at least partially unexpected, discrepancy should be clearly reported.
Intermediate level rules (examples)	
	1. When > 40% of the components of a family of cross-reactive components are positive, a positivity towards the whole family must be considered [18].
	2. Immunotherapy is more active if sensitization to one or a few genuine components is observed [5,6].
	3. More than three different families of allergens cannot be administered to the patients [30,31].
	4. According to the ratio between the genuine and cross-reactive components, different phenotypes are identified [29].
Low level rules (examples)	
	1. Very low levels of a component associated with a very high level of IgE to a cross reactive component are most likely associated with a cross reaction between the components. This positivity is trustable only if the clinical signs associated with sensitization to these low level IgE components are evident. (for example: Lep d 2 is weakly positive, and Der f 2 is strongly positive; a cross reaction, at least in the in vitro test, should be suspected based on the > 50% identities in the primary structures).
	2. When a component is negative (e.g., Amb a 1) and the extractive allergen (Ambrosia a.) is positive, if all other cross-reactive components (such as profilins, PR-10, polcalcins) are also negative, even if belonging to other allergens, a real discrepancy is reported. The frequency of this discrepancy is calculated and shown in Allergenius, as well as the median score of ImmunoCAP for this category of allergens.

### The inference engine (IE)

The IE is the core of the ES and manages the data of the hierarchical structure of the KB frames. This allows the analysis of all components (both single allergens and allergen families) using an iterative approach. Briefly, using the I/O routines, the IE acquires the patient’s ISAC results and elaborates these results to obtain further data (Table 2). The IE elaboration is based on both forward chaining rules (i.e., knowing the data, one single expected result is obtained, a data-driven reasoning) and backward chaining relations (i.e., knowing the effect, one or more pre-existing conditions are provided, a goal-driven reasoning). Starting from all these data and from the reasoning strategies implemented, the IE checks the consistency between the components, the sIgE or SPT test results and the declared allergic disease(s). In this context, a low level management of uncertain (or unknown) SPT or sIgE data are also performed. Then, the IE comments on the frequency of the sIgE profile observed, ranging from a frequent, in which the patients are sensitized to one or more components belonging to the well-defined allergen families, to more rare situations when few positive scattered components are observed.

**Table 2:** Data used or calculated by the inference engine (IE)

1) Single components, such as Der p 1 or Der p 2, in ISU.
2) Single source allergens, corresponding to the sum of the components, such as Der f 1 and Der f 2 for Dermatophagoides pt.
3) Comprehensive source allergens (such as Der f 1, Der f 2 and Der p10 – a component highly homologous to the Der f 10 that is not included in the ISAC panel).
4) Cross-reactive families (such as tropomyosins, represented by Ani s 3, Bla g 7, Der p 10 and Pen m 1), as well as PR-10 and profilins, among others.
5) Allergen groups (such as mites, grasses and trees), represented by the sum of the score of the components belonging to those groups.
6) Enlarged component groups (such as inhalants, food, latex and Hymenoptera venoms), represented by the sum of the score of the components belonging to those large groups.
7) The total amounts of the scores for either genuine or cross-reactive inhalant components are also calculated to support the patient’s phenotyping.

According to the level of the detail required, the IE adds the information related to the component characteristics, such as the resistance to physical factors and the associated risks, and evaluates the “relative level of positivity” in the context of the component family. The last issue was implemented because the specific sIgE scores for the inhalants can range between 0 and 100 ISU, while the scores for the food components generally range between 0 and 10 ISU. Unfortunately, there is only one ISAC scale (from 0 to 100 ISU). From a practical point of view, a score of 10 ISU for Der p 1 (low level) is different from a score of 10 ISU for Pru p 3 (a high level, compared to the average results). The product of the IE is a raw report that contains three different sections and subsections (Table 3).

**Table 3:** Sections of the Allergenius report

1^st^ section,	
	1. Headings.
	2. Patient’s ID, test date and methodology used.
	3. The declaration of the patient’s sensitization (and not allergy) as the unique result generated by the ES report.
	4. An overview of the patient’s sensitization (genuine, cross-reactive, inhalant, food, hymenoptera, etc.) and the frequency of such a distribution of sensitization in an uncensored population of allergic patients.
	5. Some general statistical data, such as the total ISAC score (in general correlating to the level of circulating total IgE), the fraction of the genuine and cross-reactive components, the fraction of food allergens, etc.
	6. An analysis of the compatibility between the ISAC results and the diseases suffered by the patients.
	7. The patient’s phenotyping [32]: Type I, only genuine inhalant components; Type II, genuine inhalant components with a few cross-reactive inhalant components; Type III, both genuine and cross-reactive components are represented in the Patient’s IgE profile; Type IV, the IgE profile is constituted almost exclusively of IgE to inhalant cross-reactive components; Type V, sIgE are specific for only food components. This classification seems to be predictive of the immunotherapy outcome [6].
	8. Warnings indicating the presence of sIgE directed at potentially dangerous components [8].
2^nd^ section. The list of positive components.	
	1. IgE-positive inhalant components are described. For each component, the characteristics listed in the description of the frame structure of Allergenius are reported.
	2. Positive food components are reported; in particular, the susceptibility to heat and pH is described.
	3. The list of different families of cross-reactive components, which includes many of the above-analyzed components, is reported. The IE performs an analysis of each family, indicating whether sensitization to the single component(s) or to the whole family is suspected.
3^rd^ section. This section is considered helpful to the doctors while managing patients. It includes:	
	1. Indications of the relationships between the positive molecular component(s) and known clinical syndrome(s) or associations are listed (Table 3).
	2. Some short indication of the therapy [29,30], including the removal of the allergen immunotherapies for most well-known allergens, such as mites (D1 and D2), grasses (G2 and 6), trees (T3 and T9), weeds (W1, W6, W21) and animals (E1 and E5), or medical therapies with bronchodilators, local steroids, and new-generation anti-histaminic drugs. Note that this is a beta-version and is implemented strictly for research use only.
	3. An analysis of the discrepancies between the SPT, sIgE and ISAC results to improve the reading of the complex microarray tests.

### The general strategy for ISAC result interpretation

As already mentioned, the 112 components of the ISAC have many complex relationships, and the comprehensive use of this knowledge may add significant value to the ISAC interpretation. For example, each allergen (for example, Parietaria j.), despite being represented in the ISAC by a single “genuine” component (Par j 2), is known to consist of other components (including Par j 3, a profilin, and Par j 4, a polcalcin). These cross-reactive components (belonging to other allergen sources) are present in the ISAC. Thus, in the presence of sIgE to profilins, a cross reaction with Parietaria j. profilin cannot be ruled out or, even better, should be seriously considered [33]. A positive “genuine” component is the sign of a true specific sensitization, while the negativity of the “genuine” component(s) excludes a specific sensitization. However, the positivity of other cross-reactive components belonging to other allergen sources, such as PR-10, profilins and polcalcins, must be carefully considered in the interpretation of the clinical symptoms as well as when considering the results of the SPT and sIgE results for extractive allergens. Similarly, some extractive components, such as MUXF3, Phl p 4 and Jug r 2, carry cross-reactive carbohydrate determinants (CCD), whose cross-reactivity must be ruled out before defining their significance. Of note, using MUXF3 to evaluate the CCD in ISAC is less sensitive than the same test on the standard ImmunoCAP assay.

### Insertion of hypertext links in the ES report

The I/O structure produced by the code is a .txt report file, which is suitable for further elaboration by standard word processing tools, such as MS Word. Indeed, the initial raw text file that is generated is post-processed by a Visual Basic routine that changes all of the relevant words (such as mites, birch, Der p 1), acronyms (such as nsLTP, PR-10, etc.) and diseases (mite-shrimp-syndrome) into links to the http://www.allergenius.it site. This web site contains the additional information (pictures, taxonomy, references, etc.) that was obtained during the development of the KB but was not inserted into the frames. Of note, different examples of the ES report are available on this web site.

### Validation of the expert system

First, an in silico validation was performed. The ES was loaded with totally negative or totally positive ISAC files to confirm that all routines worked appropriately. Then, to validate the relationships between the allergens, files with positive groups (such as mites, animals, profilins, etc.) were assayed. Finally, 100 random results files were generated and processed. Because, in real life, only a maximum of 15% of the components have positive results in poly-sensitized patients, these files were designed to reflect this situation. Finally, clinical validation was performed using two approaches. The first was to compare 100 results of the ES with the results of X-Plain, the specific proprietary explanation software for ISAC analysis. Then, according to Linnett [34], 10 anonymous samples from real patients were evaluated by six allergists expert in molecular analysis and co-authors of this study. Briefly, the standard ISAC report, together with the clinical picture and the results of specific IgE tests were given to the experts together with a short questionnaire. The questionnaire itams were 1) to identify the patient, 2) the methodology used and 3) to describe the frequency of similar cases in a general population of allergic patients; 4) to define genuine and cross-reacting positive components; 5) to define food and inhalant related positive components; 6) to define whether a co-sensitization and/or a cross-sensitization was detectable; 7) to define whether a compatibility between the positive components detected and the clinical picture was present; 8) to enlist any warning related to potentially dangerous positive component; 9) to enlist all positive components (food, inhalant, latex and venoms) or component families (profilins, LTPs, PR-10 etc.); 10) to identify any potential inhalant (or pollen)-food syndrome; 11) to give immunotherapeutic indications based on the Douladiris [5] and Schmid-Grendlmeier [6] indications and, finally, 12) to identify any discrepancy between the ISAC results and other results from SPT and/or specific IgE testing. The score was 1 when the ES and the expert gave the same result; the score was 0 when the expert did not answered the single question and the score was 0.5 when the expert answered wrongly the question.

## Results

The ES implemented to support the interpretation of ISAC results was originally designed with the aim of mimicking the behavior of a molecular allergy expert. Indeed, an expert is expected to do more than just discriminate between the positive and negative results. The allergist is expected to read the ISAC results while considering the role of the allergen and component families, as well as considering the relationships between the components and the cross-reactivities. If possible, information leading to the prescription of an immunotherapy and the discrepancies between the different tests is also expected.

The ES was named Allergenius ®, and the v4.4 version was used for these tests. The production of the report text file requires a few tenths of a second. The time taken to transform the raw text file into a MS Word report using Visual Basic, during which all of the relevant words become specific links, required a few seconds. According to the user’s decision, the report language can be either English or Italian, and the level of detail can be either basic or advanced. Six different example reports (in either Italian or English and with either a basic or advanced level of detail) can be found on the http://www.allergenius.it web site.

Irrespective of the level of detail, an Allergenius report starts with the items described in Table 3. The main section details all of the positive components. These include the molecule, the origin, the family, any possible cross-reactions and any warnings related to potentially dangerous components, such as lipid transfer proteins. The “relative level of positivity” for each of the positive results (as IgEs to inhalants have a range from 0 to 100 ISU, while IgEs to foods rarely exceed 10 ISU) is also calculated. The following section details the cross-reactive components, ordered in families, with the aim of accurately describing these important sources of sensitization. In this section, an attempt to discriminate the sensitization toward one or a few of the components from the sensitization to the whole family is made. On the basis of both statistical and heuristic considerations, a sensitization to > 40% of the members of the family is considered a sensitization to the whole family. Of course, this approach cannot be applied to all families of cross-reactive components available in the ISAC; for example, there are only two polcalcins (Phl p 7 and Bet v 4) and the parvalbumins (Gad c 1) are represented by a single component. Another attempt to define the “first sensitizer” was also implemented in the IE. The first sensitizer is, in general, the earliest component to which a sensitization occurs in a patient’s life. This is normally associated with the highest ISU score. The first member of each family that appears can be easily identified by studies on the allergenic march [11]. Thus, when the highest ISAC score corresponds to the member known to appear first, this can be identified as the first sensitizer in a given patient. Of course, the members of families are extremely cross-reactive. For this reason, this algorithm may, for example, indicate that Bet v 1 is the first sensitizer in regions, such as the Mediterranean area, where birch is virtually absent. However, in these environments, other sensitizers that are highly homologous to Bet v 1 components, such as the hornbeam Car b 1 and oak Que a 1 components [35,36], are very frequent.

In the third and final section, three other calculated results are available. The first is the list of syndromes or associations related to one or more of the positive components (Table 4). The second is a list of the therapeutic suggestions for positive inhalant components. This is based on the “European” approach to immunotherapy [31,32], which limits the number of allergens to be administered. Along this line, the algorithm recently proposed for pollens has also been considered [5]. Finally, the discrepancies between the SPT and/or sIgE scores and the ISAC results [10] are also discussed. These discrepancies may be a source of confusion for allergists only partially trained in molecular allergy diagnosis.

**Table 4:** Syndromes and associations related to the sensitization to allergens or components

Syndrome or Association	Allergen or component(s) involved
Mite Shrimp syndrome	Der p 10
Mugwort chamomile association	Art v 1 (?)
Birch apple syndrome	Bet v 1 – Mal d 1
Latex fruit syndrome	Hev b 6, Hev b 7
Cypress peach syndrome	LTP
Alternaria spinach syndrome	Alt a 1
Goosefoot melon association	Che a 2, Che a 3
Russian thistle saffron association	Not known
Wheat Dependent Exercise Induced Anaphylaxis (WDEIA)	ω-gliadins or a high molecular weight glutenin subunit
Pellittory pistachio association	Cross reacting proteins on SDS PAGE and immunoblot

Starting from the results obtained as described, the in silico validation was performed, and all of the tests with negative and/or positive samples had the expected results. These tests were carried out thousands of times during the development of the code, and the results were always the same.

Random results were also tested. For this, 100 random ISAC results, with a frequency of up to 15% positive components (ranging 2-17%) largely aligned with those observed in real-life patients, were obtained. Every component was present in these tests (median frequency 13%, range 5 -26%), and the ES identified all of the different components, including both the genuine and cross-reactive components and suggested the syndromes associated with the individual components with great accuracy.

**Figure 1:**
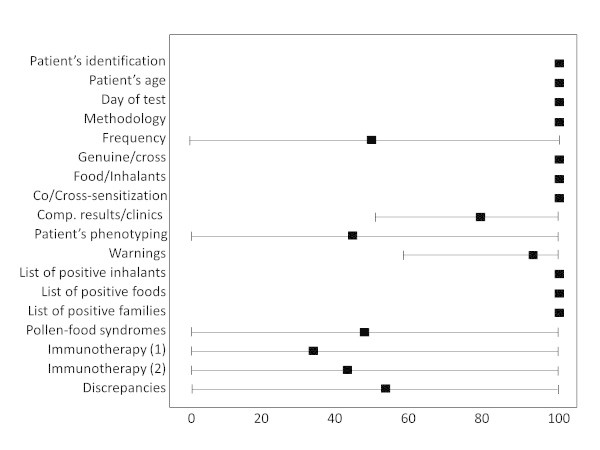
**Forest-plot of the validation results.** On the horizontal axis, the percentage of correct results observed. On the vertical axis, the different issue of the questionnaire. A black box represents the mean result for a given issue. A black box on the 100% line is indicative of “always correct” answers to the experts’ questionnaire. The lines represent the variation of the results. A line starting from 0% means that one or more expert did not answer the question. See the Materials and Methods section for a more detailed description of the parameters analyzed and of the methodology used.

The validation with X-Plain was performed on 100 ISAC results. Of these results, 15 were negative, and the remaining belonged to poly-sensitized patients. Even in this validation step, all the positive, as well as the negative, components were identified correctly, and the ES comments were almost identical to those of X-Plain. However, because of the specific structure of Allergenius, information that was unavailable using X-Plain were obtained.With regard to the human experts’ opinions, the results of the questionnaires obtained are shown in a Forest plot (Figure 1). There was a total concordance between the experts and the ES in 10/18 issues, namely identification of patients, of positive components and of characteristics of components. The compatibility of the clinic with the ISAC result and the list of warnings were considered by the experts even if few errors were made. Finally, experts had difficulties in defining the frequency of the clinical picture, the patient’s phenotyping, the pollen-food syndromes, the immunotherapeutic indications and the discrepancies between the results of ISAC and those of other sIgE tests. Notably, the experts did not identify issues that the ES was unable to detect. However this results was conditioned by the “closed” list of questions asked to the experts. Even more interestingly, only two experts were able to correctly answer all the points of the questionnaire.

The validation procedures performed herein demonstrated that the ES is highly capable of detecting every positive and negative component, of distinguishing cross- and co-sensitization and in identifying sensitization to genuine and cross-reactive components. Allergenius is able to process all possible ISAC results using two different levels of detail. The basic level, compared to the X-plain software report, adds some additional information. This includes the analysis of cross- and co-sensitization, the patient’s phenotype, the semi-quantitative and relative score of each component, the consistency between the patient’s disease and the positive component, the discrepancies between the ISAC results and other tests, the list of possible syndromes associated with the positive components and a proposal for therapy for inhalants. The advanced level adds much more statistical information to the report, such as the frequency of a given component, the median score, and the highest level observed, among others.

## Discussion

The strengths of this approach are numerous. In general, ESs are characterized by a) the consistency and traceability of the results compared to an individual’s interpretation; b) the productivity gains (less expensive than an expert); c) the performance gains (quicker than humans); and d) the availability of the solution (can be available anytime, anywhere). Moreover, using Flex, the structure of the code is in plain English and is aligned with the experts’ understanding of the real world. Finally, the hierarchical structure allows the insertion of new facts and rules without significantly modifying the program. Allergenius has other specific advantages; for example, the report language is either English or Italian, but thanks to its structure, other languages can be easily implemented, reducing the risk of misunderstanding the report. As Allergenius is an ES written by MDs for MDs, special attention was given to the construction of the report. For example, the above-mentioned level of detail can be defined by the user. Thus, experts will not be obliged to read that Der p 1 is a cysteine protease or that Dermatophagoides pt. belongs to the Pyroglyphidae family, and novices will not be confused by too much data. If needed, further information is available using a dedicated hypertext link.

Weaknesses are also present. For example, while the rules from the literature were always considered true, other rules were discussed within the group of authors: some were widely accepted, others were not. However, the rules were organized in such a way that they can be modified as soon as other or more accurate rules become available. Of course, starting from 112 different components, the number of combinations is enormous. The problems related to the IgE profiles are very complex, and the advancement of molecular allergy research is very fast. Thus, Allergenius will remain effective only under a continuous revision process. We are also aware that a significant number of colleagues dislike computer-supported diagnoses. However, relevant information, such as references, blogs, comments and mails, are often used, making computer-supported diagnosis generally accepted. The authors have extensively discussed whether the code should be freely available on the internet or should remain proprietary to the laboratory where the ISAC is carried out. The former possibility is extremely interesting; virtually all ISAC studies carried out worldwide could be analyzed using the ES. Comments, observations and errors could be noticed immediately, and the code could be improved very quickly. Even more, using elementary machine-learning techniques, the rules and the knowledge base could be updated in real time. Nevertheless, in the absence of a very exhaustive clinical validation, the risk of some unknown bugs or errors cannot be ruled out. Of course, the authors are available to receive ISAC results, along with the SPT results, specific IgE results and a brief sketch of the clinical history, to allow other colleagues to evaluate and comment on the present and future Allergenius releases. This will represent a more in-depth and rigorous validation not only of the rules implemented but also of the capacity of an ES to support the interpretation of results obtained by allergy molecular diagnostics tests.

A molecular allergy diagnosis based on microarray technology may sometimes be a complex task due to the large number of results available, the relationships between the components and the rules that govern virtually every single component. For this reason, a computer-assisted tool based on data from the literature, international guidelines and experts’ opinion may play a significant role, particularly in countries where these molecular tools are only recently available. In the present version, Allergenius is a very advanced working prototype with great possibilities of improvement. Allergenius could be useful in the interpretation of molecular allergy tests, in allergen microarray diagnostics training programs and in supporting allergists in their profession. By adopting a frame hierarchy approach, the structure based on Flex allows for real-time modification of the knowledge base as well as the adaption to specific environments (Northern or Southern countries, US or EU, children or adults, etc.).

Allergists are aware that the characteristics of the 112 different components may sometimes be difficult to keep in mind; for this reason, an ES tool could definitely be accepted by the allergist community. If not, the efforts to build this system and the results described in this work can still be useful to allergists to realize the large volume of information that can be extracted from an ISAC report.

## Abbreviations

BD: Biology doctor; CCD: Cross-reacting carbohydrate determinants; CSD: Computer-supported diagnosis; ES: Expert system; EU: European community; I/O: Input/output; ID: Identification (e.g., Patient’s ID); IE: Inference engine; ISAC: Immuno solid phase allergen chip; ISU: ISAC standard units; KB: Knowledge base; LPA: Logic programming associates; MA: Microarray; MD: Medical doctor; MS-Word: Microsoft word; nsLTP: Nonspecific lipid transfer proteins; PhD: Doctor of philosophy; PR-10: Pathogen related protein 10; RCSB: Research collaboration for structural bioinformatics; sIgE: Specific IgE; SPT: Skin prick test; US: United States; WHO/IUIS: World Health Organization/international union of immunological societies.

## Competing interests

No competing interests to declare.

## Author’s contributions

GM, wrote a large portion of the FLEX code, created the rule databases, including the components, allergens and component-associated diseases and wrote the first version of the manuscript.Clive Spenser, Logic, Marketing Director of Logic Programming Ass., London, introduced GM to the FLEX language and oversaw the construction of the hierarchical structure of the Allergenius knowledge base and the development of the input routines. GR, bio-informatics, developed the VBA routines for the elaboration of the Allergenius final reports. GP, contributed to the list of rules and revised the manuscript. EC, contributed to the list of rules and therapeutic suggestions and revised the manuscript. AR, contributed to the list of component-associated syndromes and component-related clinical pictures. AMR, contributed to the analysis of the discrepancies and revised the manuscript. EN, clinical allergist, revised, corrected and commented on the Allergenius reports and revised the manuscript. EDL, clinical allergist, revised, corrected and commented on the Allergenius reports and revised the manuscript. GWC, clinical allergist, coordinated the whole project, revised, corrected and commented on the Allergenius reports and revised/approved the manuscript. All authors read and approved the final manuscript.
